# Sodium and Its Role in Cardiovascular Disease – The Debate Continues

**DOI:** 10.3389/fendo.2016.00164

**Published:** 2016-12-23

**Authors:** Yee Wen Kong, Sara Baqar, George Jerums, Elif I. Ekinci

**Affiliations:** ^1^Department of Endocrinology, Austin Health, Heidelberg, VIC, Australia; ^2^Department of Medicine, Austin Health, The University of Melbourne, Melbourne, VIC, Australia; ^3^Menzies School of Health Research, Darwin, NT, Australia

**Keywords:** sodium intake, salt intake, dietary sodium intake, diabetes mellitus, cardiovascular disease, cardiovascular death, morbidity and mortality, chronic kidney disease

## Abstract

Guidelines have recommended significant reductions in dietary sodium intake to improve cardiovascular health. However, these dietary sodium intake recommendations have been questioned as emerging evidence has shown that there is a higher risk of cardiovascular disease with a low sodium diet, including in individuals with type 2 diabetes. This may be related to the other pleotropic effects of dietary sodium intake. Therefore, despite recent review of dietary sodium intake guidelines by multiple organizations, including the dietary guidelines for Americans, American Diabetes Association, and American Heart Association, concerns about the impact of the degree of sodium restriction on cardiovascular health continue to be raised. This literature review examines the effects of dietary sodium intake on factors contributing to cardiovascular health, including left ventricular hypertrophy, heart rate, albuminuria, rennin–angiotensin–aldosterone system activation, serum lipids, insulin sensitivity, sympathetic nervous system activation, endothelial function, and immune function. In the last part of this review, the association between dietary sodium intake and cardiovascular outcomes, especially in individuals with diabetes, is explored. Given the increased risk of cardiovascular disease in individuals with diabetes and the increasing incidence of diabetes worldwide, this review is important in summarizing the recent evidence regarding the effects of dietary sodium intake on cardiovascular health, especially in this population.

## Introduction

High dietary sodium intake has been related to high blood pressure for more than 4,000 years (1). The concept that fluid volume influenced arterial pressure was then deduced by Stephan Hales in the early 18th century. He provided the scientific rationale that sodium intake might be related to blood pressure since blood volume is largely determined by its sodium and water content (2). Over the next two centuries, other investigators including Ambard and Beaujard, demonstrated that high sodium intake contributes to high blood pressure in both humans and animals (1). The notion of sodium restriction potentially lowering blood pressure was supported by epidemiological observational studies of communities with habitual low sodium intake (3, 4). In communities with low sodium intake, blood pressure tended to be lower and did not rise with age. This led to the hypothesis that, at a population level, blood pressure may be correlated with sodium intake (3, 4). Since this early body of work, there have been many epidemiological and experimental studies confirming the association between high sodium intake and high blood pressure. As elevated blood pressure was associated with increased risk of cardiovascular disease (5), it was hypothesized that high dietary sodium intake may be associated with increased cardiovascular morbidity and mortality (5, 6).

Based on this body of evidence, multiple dietary guidelines for sodium intake have been published (Table 1). The 2010 dietary guidelines for Americans recommended sodium intake to be less than 2,300 mg/day (100 mmol/24 h) for the general population and less than 1,500 mg/day (65 mmol/24 h) for higher risk subgroups who are at least 51 years old or African-Americans or have hypertension, diabetes, or chronic kidney disease (7). However, the American Heart Association (AHA) 2010 guidelines contended this and recommended for sodium intake to be less than 1,500 mg/day (65 mmol/24 h) for the entire U.S. population (8). On the other hand, the World Health Organization (WHO) 2012 guidelines recommended a sodium intake of less than 2,000 mg/day (87 mmol/24 h) for adults (9). The American Diabetes Association (ADA) also released a statement in 2008 recommending sodium intake to be less than 2,300 mg/day (100 mmol/24 h) in individuals with hypertension or normotension and less than 2,000 mg/day (87 mmol/24 h) for individuals with diabetes and symptomatic heart failure (10). Furthermore, the Kidney Disease: Improving Global Outcomes (KDIGO) 2012 international guidelines suggested a sodium intake of less than 90 mmol/24 h to prevent progression of chronic renal disease in adults (11).

**Table 1:** **Summary of guidelines for dietary sodium intake over time**.

Year	Summary of guidelines
2008	ADA: Normotension, HTN: <100 mmol/24 h DM, symptomatic HF: <86 mmol/24 h
2010	HHS and USDA: General population: <100 mmol/24 h Age ≥51, African-American HTN, DM, and CKD: <65 mmol/24 h
	AHA: <65 mmol/24 h for entire U.S. population
2012	WHO: <86 mmol/24 h
	KDIGO: <90 mmol/24 h
2013^a^	ADA: <100 mmol/24 h, further reductions on individual basis
	AHA: ideally <65 mmol/24 h
	NHMRC: ideally <70 mmol/24 h
2014	ASH and ISH: reduce sodium intake, but no target level
2015	HHS and USDA: <100 mmol/24 h

*ADA, American Diabetes Association; AHA, American Heart Association; WHO, World Health Organization; KDIGO, Kidney Disease: Improving Global Outcomes; HHS and USDA, U.S. Department of Health and Human Services and U.S. Department of Agriculture; ASH and ISH, American Society of Hypertension and International Society of Hypertension; NHMRC, National Health and Medical Research Council. HTN, hypertension; DM, diabetes mellitus; HF, heart failure; CKD, chronic kidney disease; BP, blood pressure*.

*^a^IOM: no clear evidence showing that guidelines for sodium intake <100 mmol/24 h is beneficial or harmful. No evidence that subgroups should have different sodium intake guidelines*.

However, low dietary sodium intake has pleotropic effects, which could contribute to cardiovascular health. Therefore, rationalizing that low sodium intake reduces adverse cardiovascular outcomes based on its blood pressure lowering effects alone may not be appropriate. In 2013, the Institute of Medicine (IOM) in the U.S. examined the evidence on the effect of dietary sodium intake on health outcomes in the U.S. general population and higher risk subgroups (12). It was concluded that there was a lack of clear scientific evidence showing benefit or harm of reducing sodium intake to recommended levels (<100 mmol/24 h) (12). There was also limited evidence supporting different dietary sodium guidelines for higher risk subgroups (12). Since then, guidelines for dietary sodium intake have been revised. The 2015–2020 dietary guidelines for Americans now recommend sodium intake to be less than 2,300 mg/day (100 mmol/24 h) (13). The ADA supports this and also comments that further reductions in sodium intake need to be considered on an individual basis for those with diabetes and hypertension (14). In addition, the AHA 2013 guidelines now specify that their current sodium intake recommendations of no more than 2,400 mg/day (104 mmol/24 h) and ideally less than 1,500 mg/day (65 mmol/24 h) are targeted toward reducing blood pressure (15). The American Society of Hypertension (ASH) and International Society of Hypertension (ISH) also suggest reducing sodium intake but do not recommend a target level (16).

In Australia, the National Health and Medical Research Council (NHMRC) recommended sodium intake to be ideally less than 1,600 mg/day (70 mmol/24 h) and at a maximum of 2,300 mg/day (100 mmol/24 h) for adults in 2013 (17). This recommendation was supported by the National Heart Foundation of Australia who also suggested for sodium intake to be less than 2,300 mg/day (100 mmol/24 h) for adults and 1,600 mg/day (70 mmol/24 h) for those with hypertension (18).

## Materials and Methods

A literature search in MEDLINE (1946–July 2016) was performed using a combination of the following search terms: salt, salt intake, dietary salt intake, dietary sodium intake, dietary sodium, dietary sodium chloride (adverse effects, antagonists, and inhibitors, urine), hypertension, heart rate, immune system, cardiovascular, cardiovascular disease, cardiovascular mortality, mortality, diabetes, type 2 diabetes, and type 1 diabetes. Combinations of limitations including English language, core clinical journals, and journal article were placed on the search terms. References from the relevant papers were also sourced.

## Discussion

### Effects of Sodium Intake on Blood Pressure

Recommendations to reduce sodium intake have been based on the prevailing view that high sodium intake is detrimental to blood pressure, which is a surrogate endpoint for cardiovascular morbidity and mortality (19). There is overwhelming evidence to support that higher sodium intake is associated with elevated blood pressure (1, 3, 4, 6, 20–23). This is consistent in both experimental animal models and human studies (6). Conversely, sodium restriction is associated with reduced blood pressure (6, 22, 23). Lower sodium intake, however, may have pleotropic effects (Table 2).

**Table 2:** **Favorable versus unfavorable effects of reduced dietary sodium intake**.

Favorable effects	Unfavorable effects
↓ Blood pressure	↑ Cholesterol
↓ Left ventricular hypertrophy	↑ Catecholamines
↑ Antiproteinuric effect of drugs for albuminuria	↑ Renin–angiotensin–aldosterone system activation
↓ Pro-inflammatory state	

### Effects of Sodium on Cardiovascular Health

Over the recent years, dietary sodium has been shown to have other effects such as impacting on rennin–angiotensin–aldosterone system, left ventricular hypertrophy, heart rate, albuminuria (microalbuminuria/proteinuria), insulin sensitivity, lipids, immune function, endothelial dysfunction, and sympathetic nervous system activity.

However, studies demonstrating the effects of dietary sodium on these factors have demonstrated inconsistent results. The discrepancy in results may be attributed to methodological differences among studies. This includes differences in the methods of measurement and ranges of dietary sodium intake, study populations, study outcomes, and failure to explore non-linear associations (24). Many of the methodological controversies pertain to the accuracy in measuring dietary sodium intake (25). Twenty-four hours urine collection is considered the gold standard method for estimating sodium intake because approximately >90% of ingested sodium is excreted in the urine in healthy individuals (25). However, we have previously demonstrated that the intraindividual day-to-day variability of a single 24-h urine collection is approximately 20% (26). As such, averaging multiple 24-h urine collections to minimize random error from day-to-day variability in sodium intake provides the most accurate estimation of an individual’s usual sodium intake (25). This is dependent on measures to identify and reduce under-collection or over-collection of these 24-h urine collections (25).

However, many studies estimate dietary sodium intake through dietary surveys or overnight and spot urine collections due to the lower burden on participants (25). Dietary surveys tend to underestimate dietary sodium intake by 30–50% due to underreporting, difficulty in measuring discretionary sodium use at the table and in cooking, and incomplete food composition databases (25, 26). Moreover, food composition databases can vary greatly in their approximations of the nutrient content in foods, depending on the food manufacturers, the methods in measuring nutrient content, natural variations in food composition, and frequency of updates to the food databases (27). This can further compound the inaccuracy in sodium intake estimations in dietary surveys. In addition, the validity of different dietary assessment tools is variable due to their limitations. Twenty-four hours dietary recalls do not reflect long-term dietary patterns and a single 24-h dietary recall does not account for daily variability in dietary intake (25). Conversely, food frequency questionnaires have the potential for recall bias (25). Therefore, the degree of imprecision in estimating dietary sodium intake can also be contributed by the choice of dietary assessment tool.

On the other hand, overnight and spot urine collections are weak surrogates for 24-h urine collections because they have not been sufficiently validated and could underestimate or overestimate 24-h urinary sodium excretion (25, 28). Although several formulae have been proposed to reliably approximate 24-h urinary sodium excretions with overnight and spot urine collections, this has been controversial because overnight and spot urine collections can vary with different genders, ethnic groups, hydration status, and duration and volume of urine collection (28). Moreover, urinary sodium excretion is also affected by diurnal variation (25). Therefore, overnight and spot urine collections are unlikely to be satisfactory substitutes for 24-h urine collections in estimating an individual’s sodium intake.

In addition to the method used to estimate dietary sodium intake, the inconsistency in results could also be attributed to limitations of study design. Since many of the studies, which showed the effect of sodium intake on cardiovascular health are observational studies, they are susceptible to confounders and reverse causation. Potential confounders can be reduced in observational studies through restricting or matching participants and performing stratified or multivariate analysis (29). However, this is not applicable to unknown confounding variables, which can distort the association between the exposure and outcome of the study (29). In addition, observational studies are susceptible to reverse causation (29). Reverse causation tends to occur in studies that involve individuals with pre-existing cardiovascular morbidity and cardiovascular risk factors. These individuals may be more likely to restrict their sodium intake because of their comorbidities, which can create an association between lower sodium intake and increased cardiovascular morbidity and mortality. However, the increased cardiovascular morbidity and mortality may not be due to lower sodium intake. Conversely, it may be that lower sodium intake is a result of having pre-existing cardiovascular morbidity and risk factors. Therefore, caution is required in the interpretation of causal associations between exposure and outcome in observational studies.

In addition, there is a lack of consistency in definitions of “low,” “moderate,” and “high” dietary sodium intake and “severe” and “moderate” sodium restriction. For example, many studies used the term “moderate sodium restriction” when the mean or median reduction in dietary sodium intake is less than 120 mmol/24 h. However, Grassi et al. used this term despite having a 140 mmol/24 h reduction in sodium intake (mean sodium reduction 129–136 mmol/24 h) (30). Conversely, Ferrara et al. (31) used the term “severe sodium restriction” when there is only a mean sodium reduction of 63–75 mmol/24 h. This highlights the importance of having consistent definitions of these terms to facilitate the interpretation of study results. In this review, these terms will be defined based on majority of the studies. Low, moderate, and high dietary sodium intake is defined as less than 120 mmol/24 h, 120–150 mmol/24 h, and more than 150 mmol/24 h, respectively. Severe sodium restriction is defined as having mean or median sodium reduction of at least 120 mmol/24 h while moderate sodium restriction is defined as mean or median sodium reduction of less than 120 mmol/24 h.

### Effects of Sodium Intake on Left Ventricular Hypertrophy

Higher sodium intake was proposed to be associated with left ventricular hypertrophy (32–35), which is an independent predictor of cardiovascular morbidity and mortality (36). In both individuals with normotension and those with untreated essential hypertension, there was a correlation between higher left ventricular mass and higher urinary sodium excretion reflective of a higher sodium intake (32). This substantiated the findings of another study highlighting that dietary sodium intake was the best predictor of the degree of left ventricular hypertrophy in individuals with essential hypertension (34). In addition, Kupari et al. (33) observed that in a random sample of subjects born in 1954 with both systolic blood pressure and sodium intake above the population median, left ventricular mass was the highest. It was suggested that high sodium intake sensitized the heart to the hypertrophic stimulus of pressure load, which could result in the synergistic interaction of dietary sodium intake with blood pressure on left ventricular mass (33). On the other hand, moderate sodium intake restriction (mean sodium reduction of 63–75 mmol/24 h for 6 weeks) in men with inadequately controlled primary hypertension significantly reduced blood pressure and was associated with reductions in left ventricular mass to the same degree as thiazide diuretic therapy (31).

### Effects of Sodium Intake on Heart Rate

Increased heart rate was demonstrated to be independently associated with increased cardiovascular and all-cause mortality (37, 38). This may be attributed to its effect on the diastolic period, which is important for the myocardial perfusion of the left ventricle (39). In addition, the long-term load on both the left ventricle and systemic arteries had been proposed to be related to the product of heart rate and systolic blood pressure (39). Therefore, heart rate may play a more important role in cardiovascular health than anticipated. However, the effects of dietary sodium on heart rate have been frequently overlooked in favor of its effects on blood pressure (39). Studies that demonstrated the effects of dietary sodium on heart rate have shown conflicting results. Graudal et al. (40) observed that sodium restriction (sodium reduction of 42–341 mmol/24 h in studies lasting 4–90 days) was independently associated with increased heart rate in healthy individuals and individuals with hypertension. Higher heart rate associated with lower sodium intake may contribute to higher cardiovascular morbidity and mortality. Although a few randomized controlled trials showed a possible dose–response relationship between reduced dietary sodium and increased heart rate, the data were insufficient for a reliable conclusion (40). In contrast, high sodium intake (250 mmol/24 h for 7 days) was associated with reduced mean 24-h heart rate in individuals with mild-to-moderate essential hypertension (41). This was also observed in sodium-resistant individuals with hypertension or normotension (sodium intake of up to 300 mmol/24 h) (39) and healthy normotensive individuals (sodium intake of 154 mmol/24 h for 7 days) (42). However, a few studies have demonstrated no significant change in heart rate with high sodium intake (305 mmol/24 h for 7 days) in normotensive individuals (43) or low sodium intake (sodium intake of 80 mmol/24 h for 8 weeks) in individuals with untreated mild-to-moderate essential hypertension (30). Given the discrepancy in results, more studies in this area are required to investigate the relationship between dietary sodium intake and heart rate.

### Effects of Sodium Intake on Albuminuria

Albuminuria is known to be an established risk factor for cardiovascular disease, especially in individuals with diabetes (44, 45). This risk increases across the range of urinary albumin excretion, including within the normal range (44). Epidemiological studies demonstrated that increased sodium intake was independently associated with increased urinary albumin excretion (46, 47). In individuals with type 1 diabetes, higher dietary sodium intake may be associated with microalbuminuria, especially in overweight individuals (48). This was supported by a randomized controlled trial showing that individuals with type 2 diabetes and microalbuminuria had a greater increase in blood pressure and increase in albumin excretion ratio during high sodium intake (250 mmol/24 h for 7 days) (49). This was associated with insulin resistance, indicating that insulin resistance could contribute to increased sodium sensitivity of blood pressure and albuminuria (49). The association between higher sodium intake and increased albuminuria was also demonstrated by another study in 270 individuals with type 2 diabetes (50). However, this study showed a reverse *J*-shaped relationship between sodium intake and albuminuria, where both lower (sodium intake of <170 mmol/24 h) and higher (sodium intake of >203 mmol/24 h) dietary sodium intake were associated with higher urinary albumin excretion (50). On the contrary, Horikawa et al. (51) reported that there was no significant association between overt nephropathy and sodium intake in Japanese individuals with type 2 diabetes aged 40–70 years old. In contrast, blood pressure and urine protein excretion were reduced during modest sodium restriction (mean sodium reduction of 78 mmol/24 h for 4 weeks) in black individuals with hypertension (52). The discrepancies among results from different studies suggest that further investigations are required to establish the effects of dietary sodium intake on albuminuria.

In addition, sodium restriction was shown to potentiate the antiproteinuric effect of drugs used to treat albuminuria (53). These drugs include angiotensin-converting enzyme inhibitors and angiotensin receptor blockers (54). They reduce albuminuria by blocking rennin–angiotensin–aldosterone system in individuals with type 2 diabetes, thereby reducing cardiovascular risk and nephropathy (45). We have reported that the antiproteinuric and antihypertensive effects of angiotensin receptor blockers (losartan) were increased during low sodium intake (mean sodium intake of 80–85 mmol/24 h) in individuals with type 2 diabetes, hypertension, and albuminuria (53). We have also demonstrated that increased sodium intake through sodium supplementation (100 mmol/2h) reduced the antialbuminuric effect of angiotensin receptor blockers (telmisartan) with or without hydrochlorothiazide in individuals with hypertension and type 2 diabetes (55). However, it was observed that this blunting effect was only in individuals with habitual low sodium intake (24-h urinary sodium excretion of <100 mmol/24 h). In individuals with suppressed rennin–angiotensin–aldosterone system due to habitual high sodium intake (24-h urinary sodium excretion of >200 mmol/24 h), increased sodium intake does not alter the response to angiotensin receptor blockers (55). Therefore, this suggested that renal albumin excretion can be modulated by dietary sodium intake when the rennin–angiotensin–aldosterone system is not suppressed by habitual low sodium intake, but is not responsive to further increases in dietary sodium intake when the rennin–angiotensin–aldosterone system is suppressed by habitual high sodium intake (55, 56). Additionally, angiotensin receptor blockers reduced the relative risk of renal and cardiovascular events to a greater extent during lower dietary sodium intake in individuals with type 2 diabetes complicated by nephropathy (57). In contrast, higher sodium intake attenuated the renal and cardiovascular protective effects of angiotensin receptor blockers in these individuals (57). This was supported by a study, which showed that the antiproteinuric effect of angiotensin-converting enzyme inhibitors (lisinopril) was abolished with high sodium intake (200 mmol/24 h) and was restored with sodium restriction (sodium intake of 50 mmol/24 h) in individuals with proteinuria aged 26–56 years old (58).

### Effects of Sodium Intake on Renin–Angiotensin–Aldosterone System

The rennin–angiotensin–aldosterone system evolved over time to maintain sodium and body volume homeostasis (59). This system is therefore important in maintaining sodium and fluid balance during reduced sodium or reduced fluid intake (60). Physiologic compensatory activation of the rennin–angiotensin–aldosterone system may occur during sodium restriction (61). Activation of the rennin–angiotensin–aldosterone system contributes to increased cardiovascular morbidity and mortality (62). Plasma renin activity has been suggested as a surrogate marker of rennin–angiotensin–aldosterone system activation and high plasma renin activity was demonstrated to be an independent predictor of major vascular events and cardiovascular mortality in a population of high-risk individuals with atherosclerosis and/or diabetes (63). This suggests that blockade of the renin–angiotensin–aldosterone system may be beneficial for cardiovascular health. However, studies have shown that the aldosterone escape phenomenon can occur in some individuals during long-term blockade of the renin–angiotensin–aldosterone system (64–66). This phenomenon is characterized by increases in plasma aldosterone levels after the initial reduction or lack of change in aldosterone levels with renin–angiotensin–aldosterone system blockade (64). This may be more pronounced in individuals on a sodium restricted diet (66). Therefore, the renoprotective effect of renin–angiotensin–aldosterone system blockade may be reduced in these individuals with lower sodium intake. Despite recognized benefits of renin–angiotensin–aldosterone system blockade in individuals with diabetes, a greater reduction in dietary sodium intake is associated with an increased risk of developing aldosterone escape (66), which may be associated with increased cardiovascular (67) and renal morbidity (64). In individuals with type 1 diabetes and diabetic nephropathy, the degree of aldosterone escape was observed to be associated with a greater decline in glomerular filtration rate (64). Therefore, individuals with diabetes on sodium restriction may require additional aldosterone blockade to achieve optimal renoprotection (64, 65). Hence, dietary sodium restriction may not be appropriate in all individuals.

In individuals with mild-to-moderate hypertension, a high renin–sodium profile before and after antihypertensive treatment was independently associated with a higher subsequent risk of myocardial infarction (68, 69). He et al. (70) showed that plasma renin activity and plasma aldosterone increased during acute severe sodium restriction (5 days) in individuals with hypertension (mean sodium reduction of 293 mmol/24 h) and individuals with normotension (mean sodium reduction of 266 mmol/24 h). In contrast, He and MacGregor (23) reported that there was only a small increase in plasma renin activity and plasma aldosterone with modest sodium restriction over a longer period (≥4 weeks) in individuals with hypertension (median 24-h urinary sodium reduction of 78 mmol/24 h) and individuals with normotension (median 24-h urinary sodium reduction of 74 mmol/24 h). However, in a meta-analysis of individuals with hypertension or normotension, sodium restriction was shown to significantly increase plasma renin and aldosterone in proportion to the decrease in sodium intake, even in studies with longer duration (≥4 weeks) of moderate sodium restriction (sodium reduction of <100 mmol/24 h) (22). This suggested that the acute increase in plasma renin and aldosterone might persist if sodium restriction was maintained (22). This was further supported by a more recent meta-analysis, which demonstrated that low sodium intake (<120 mmol/24 h) was associated with significant increases in plasma renin and aldosterone, including in studies with a longer period of sodium restriction (≥4 weeks) (71). This discrepancy may be explained by the difference in the degree and period of sodium restriction because maximum stimulation of renin–angiotensin–aldosterone system occurred during prolonged very low sodium intake (61). In a cross-sectional study, we have demonstrated that in individuals with type 1 and type 2 diabetes, lower 24-h urinary sodium excretion was associated with higher serum aldosterone (72). This was more prominent in those who were not taking medications that would interfere with the renin–angiotensin–aldosterone system. However, we could not detect such a relationship between plasma renin activity and 24-h urinary sodium excretion, which could be partly attributed to the overall reduced plasma renin activity in individuals with diabetes (72). Conversely, in an interventional study, we reported that in individuals with hypertension and type 2 diabetes, plasma renin activity level was significantly higher with habitual “low” sodium intake (mean 24-h urinary sodium excretion of 126 mmol/24 h) than with habitual high sodium intake (mean 24-h urinary sodium excretion of 256 mmol/24 h) (73). We have demonstrated that short-term sodium supplementation (100 mmol/24 h) led to a significant reduction in the angiotensin receptor blockers-induced increase in plasma renin activity and a trend toward blunting of the angiotensin receptor blocker-induced increase in serum aldosterone in individuals with type 2 diabetes (73).

### Effects of Sodium Intake on Lipids

Moderate to severe sodium restriction has adverse effects on serum lipids (22, 74, 75). Since the risk of cardiovascular disease increases in proportion to serum lipid levels (76), the adverse effects of sodium restriction on serum lipids could contribute to increased cardiovascular risk. Studies demonstrated that total cholesterol and low density lipoprotein cholesterol increased significantly with short-term low sodium intake (20 mmol/24 h for 1 week) in non-obese normotensive individuals aged 19–78 years old (74) and in healthy men (75). Graudal et al. (22) observed increased total cholesterol and low density lipoprotein cholesterol levels without changes in high-density lipoprotein cholesterol and triglycerides mainly in studies with short-term large reductions in sodium intake (sodium reduction of >100 mmol/24 h for <4 weeks). However, a few studies with long-term moderate sodium restriction (mean sodium reduction of 75 mmol/24 h for >4 weeks) in the meta-analysis reported that the effect of sodium restriction on lipids was not statistically significant (22). This suggested that total cholesterol and low density lipoprotein cholesterol were increased during short-term severe sodium restriction, but there were no significant changes in serum lipid levels during long-term moderate sodium restriction in studies with individuals with hypertension or normotension (22). Moreover, another meta-analysis of studies in individuals with hypertension or normotension also demonstrated that increased cholesterol and triglycerides during moderate sodium restriction (median sodium reduction of 81 mmol/24 h) were in short-term studies (<2 weeks), with no statistical significance in long-term studies (≥4 weeks) (71). This was substantiated by studies showing that moderate sodium intake over a longer period did not affect serum lipid concentrations in non-obese normotensive individuals (sodium reduction of 115 mmol/24 h) (77) and individuals with mild-to-moderate hypertension (mean 24-h urinary sodium reduction of 52 mmol/24 h) (78). In addition, a meta-analysis of long-term randomized controlled trials (≥4 weeks) reported that sodium restriction had no significant effect on serum lipid levels in adults (79). Therefore, this suggested that the extent and duration of sodium restriction could influence its effect on lipid levels.

### Effects of Sodium Intake on Glucose Metabolism

Dietary sodium restriction has also been suggested to adversely affect glucose metabolism and decrease insulin sensitivity (74, 80). In addition, its activation of the renin–angiotensin–aldosterone system (61) and sympathetic nervous system (22, 71, 74) may further reduce insulin sensitivity (81–83). The renin–angiotensin–aldosterone system has been shown to predominantly mediate reduced insulin sensitivity through angiotensin II (82). Garg et al. (84) reported that short-term severe salt restriction (24-h urinary sodium excretion of <20 mmol/24 h for 7 days) was independently associated with increased insulin resistance in healthy individuals. However, although plasma renin activity, angiotensin II levels, 24-h urine aldosterone, and 24-h urine noradrenaline excretion were also increased during low sodium intake, there were no significant correlations with the increase in insulin resistance (84). This may be related to the small sample size in some studies and differences in the methods used to assess renin–angiotensin-aldosterone system and sympathetic nervous system activity. When the insulin-sensitive target tissues, such as skeletal muscle, are less responsive to insulin-mediated glucose uptake, more insulin secretion is required (80). Therefore, it was proposed that the reduced insulin sensitivity during sodium restriction could contribute to hyperinsulinism (74, 80), which can in turn induce insulin resistance (85) and is associated with cardiovascular disease and type 2 diabetes (78). One study observed that in non-obese normotensive individuals aged 19–78 years old, serum insulin was significantly increased during short-term low sodium intake (20 mmol/24 h for 1 week), indicating impaired glucose metabolism (74). Another study supported this by demonstrating that insulin-mediated glucose disposal during euglycemic clamp conditions was lower with short-term low sodium intake in normotensive individuals. This showed that insulin sensitivity was reduced during short-term low sodium intake (sodium intake of 20 mmol/24 h for 6 days) (80). However, Luther et al. (60) reported that glucose-stimulated insulin secretion was reduced without affecting insulin sensitivity during short-term low sodium intake (20 mmol/24 h for 7 days) in normotensive individuals without diabetes. This discrepancy in results could be attributed to methodological differences in measuring outcomes. Therefore, more trials using consistent methods to measure glucose metabolism or insulin sensitivity are required to investigate the effect of low sodium intake on insulin sensitivity. Meland et al. (78) reported that long-term moderate sodium intake (mean 24-h urinary sodium excretion of 125 mmol/24 h for 8 weeks) did not affect insulin sensitivity since fasting insulin, insulin C-peptide, and serum glucose levels were unchanged in individuals with mild-to-moderate hypertension. Therefore, it was suggested that the effect of sodium intake on insulin sensitivity could be related to the degree and period of reduced sodium intake. On the other hand, high sodium intake improved insulin sensitivity (80), especially in individuals with diabetes (86). During high sodium intake (200 mmol/24 h for 6 days) in healthy lean normotensive individuals, the insulin-mediated glucose disposal during euglycemic clamp conditions was increased, indicating increased insulin sensitivity (80). This was substantiated by another study demonstrating that sodium loading with 8 g of salt a day (136 mmol/24 h) to achieve high sodium intake (24-h urinary sodium of 252 mmol/24 h) reduced the glycemic and insulinemic response to glucose in individuals with hypertension and type 2 diabetes (86). This showed that glucose tolerance and insulin resistance could be improved with sodium supplementation.

### Effects of Sodium Intake on Sympathetic Nervous System Activity

Sodium restriction also leads to the compensatory stimulation of the sympathetic nervous system (22, 71, 74), which has multiple adverse effects on the cardiovascular system, including left ventricular hypertrophy progression, vascular remodeling, arterial stiffness, and atherosclerosis (87). This could lead to increased cardiovascular risk. In individuals with hypertension or normotension, low sodium intake (<120 mmol/24 h) was associated with increased plasma adrenaline and noradrenaline (71). In addition, Graudal et al. (22) reported that the increase in noradrenaline was observed mainly in short-term studies (<4 weeks). This was supported by a study demonstrating an increase in plasma noradrenaline concentration during short-term low sodium intake (20 mmol/24 h for 1 week) in non-obese normotensive individuals aged 19–78 years old (74). However, Grassi et al. (30) showed that in individuals with untreated essential hypertension, a low sodium intake of 80 mmol/24 h increased sympathetic stimulation and this effect was maintained despite ongoing sodium restriction for 8 weeks. In contrast, a meta-analysis of randomized controlled trials demonstrated that there was no change in catecholamines and sympathetic tone with long-term moderate sodium restriction in individuals with hypertension (median 24-h urinary sodium reduction of 78 mmol/24 h) and individuals with normotension (median 24-h urinary sodium reduction of 74 mmol/24 h) (23). This was substantiated by a meta-analysis of long-term randomized controlled trials (≥4 weeks) showing that sodium restriction had no effect on urinary and plasma adrenaline and noradrenaline (79). The inconsistency in results was suggested to be related to the extent and duration of sodium restriction. In short-term severe sodium restriction (median of 7 days, mean sodium reduction of 196 mmol/24 h), there were significant increases in noradrenaline (22). In contrast, there was no such significant change in noradrenaline in long-term moderate sodium restriction (median sodium reduction of 78 mmol for a median of 6 weeks in individuals with hypertension, median sodium reduction of 74 mmol/24 h for a median of 4 weeks in individuals with normotension) (23). Despite the association between the increase in muscle sympathetic nerve activity and concomitant increase in plasma noradrenaline during sodium restriction (30), the discrepancy in results could also be explained by methodological differences. Whereas most studies assessed sympathetic stimulation *via* plasma and/or urinary catecholamines (79), Grassi et al. (30) measured sympathetic stimulation *via* muscle sympathetic nerve activity (microneurography), which is considered the gold standard method for assessing sympathetic outflow in humans (88). This highlights that more trials are required to elucidate the association between low dietary sodium intake and sympathetic nervous system activity.

### Effects of Sodium Intake on Vascular Endothelial Function

Vascular endothelial dysfunction has been proposed to contribute to the development of atherosclerosis (89), which is involved in the pathogenesis of cardiovascular disease (90). In recent decades, endothelial dysfunction was demonstrated to be associated with high sodium intake in both animal models and humans (89). Since endothelial dysfunction was shown to be predictive of future cardiovascular events (89), it was proposed that high sodium intake could contribute to increased risk of cardiovascular disease. In normotensive Sprague–Dawley rats on a high sodium diet for 4–5 weeks, arteriolar responsiveness to endothelium-dependent vasodilation induced by acetylcholine was decreased during high sodium intake (91). This was attributed to impaired microvascular endothelial function since responsiveness of vascular smooth muscle to nitric oxide was unaffected by high sodium intake. It was suggested that this was related to the stimulation of increased oxidant levels by high sodium intake through increased generation of reactive oxygen species in the microvascular endothelium (91). A study suggested that the increased generation of reactive oxygen species could be partly due to increased activity of NAD(P)H oxidase and xanthine oxidase, which are oxidant enzymes that produce superoxide anions (92). It was hypothesized that reactive oxygen species could contribute to reduced bioavailability of nitric oxide since the half-life of nitric oxide is reduced when superoxide anions are present (92). Given that nitric oxide plays an important role in vascular function by promoting vasodilation and inhibiting platelet and leukocyte activation (90), reduced nitric oxide bioavailability could contribute to impaired endothelial function in the microvasculature during high sodium intake (91) and may therefore contribute to the pathogenesis of atherosclerosis.

However, other studies demonstrated that low sodium intake was associated with endothelial dysfunction (93–95). Tikellis et al. (94) observed that 6 weeks of low sodium diet was associated with a fourfold increase in plaque accumulation in the aorta, increased vascular inflammation, and renin–angiotensin–aldosterone system activity in atherosclerosis-prone apolipoprotein E knockout mice. Diabetic apolipoprotein E knockout mice were also reported to have increased plaque accumulation, vascular inflammation, and renin–angiotensin–aldosterone system activity after 6 weeks of a low sodium diet (95). Conversely, a high sodium diet attenuated plaque accumulation and reduced renin–angiotensin–aldosterone system activity in the diabetic apolipoprotein E knockout mice (95). In dogs on a low sodium diet for 2 weeks, a 60% reduction in flow-induced dilation in coronary arteries was observed (93). Huang et al. (93) proposed that the associated increase in plasma angiotensin II levels during the low sodium diet induced increased activation of protein kinase C, which upregulated vascular NAD(P)H oxidase to produce superoxide and reduce nitric oxide bioavailability. This may explain why the low sodium diet impaired endothelial response to shear stress (93).

The discrepancy in findings in animal studies was also seen in studies in humans (96, 97). During sodium loading (200 mmol/24 h for 5 days) in young healthy normotensive men on a low-salt diet, Tzemos et al. (97) observed that the acetylcholine-induced endothelium-dependent vasodilation was reduced, indicating a reduction in the stimulated release of nitric oxide from the endothelium. In addition, there was reduced endothelium-dependent vasoconstriction induced by NG-monomethyl-l-arginine (l-NMMA), which indicated that the inhibition of basal release of endothelium-derived nitric oxide was reduced (97). This showed that vascular endothelial function was impaired during short-term high salt intake (24-h urinary sodium excretion of 225 mmol/24 h, 5 days) (97). However, since systolic blood pressure was increased in this study (97), it would be difficult to distinguish the adverse effect of increased sodium intake on endothelial function from that of increased blood pressure. DuPont et al. (96) separated the effect of high sodium intake from that of increased blood pressure by investigating endothelium-dependent dilation in healthy sodium-resistant individuals, who have a change of 5 mmHg or less in 24-h mean arterial pressure between low and high sodium diets. It was observed that high sodium intake (300–350 mmol/24 h) reduced endothelium-dependent dilation (96). Since endothelium-independent dilation was not affected by high sodium intake, it demonstrated that there was no change in vascular smooth muscle responsiveness. Therefore, the reduced endothelium-dependent dilation during high sodium intake was attributed to impaired endothelial function (96). Conversely, sodium restriction improved endothelial function (98). It was reported that an acute increase in flow-mediated dilation was observed after 2 days of moderate sodium restriction (24-h urinary sodium reduction of 42 mmol/24 h) in obese and overweight individuals. This was sustained even after prolonged sodium restriction (6 weeks) (98). Moreover, there was an association between a greater increase in flow-mediated dilation and a greater decrease in 24-h urinary sodium to creatinine ratio (98). Therefore, this indicated that long-term moderate sodium restriction improved endothelial function (98). This was supported by another study demonstrating that moderate sodium restriction (sodium reduction of 80 mmol/24 h for 4 weeks) improved both macrovascular (conduit arteries) and microvascular (resistance vessels) endothelial function in middle-aged and older adults with moderately elevated systolic blood pressure (99). It was proposed that this could be related to increased nitric oxide and tetrahydrobiopterin (BH_4_) bioavailability and reduced oxidative stress during sodium restriction (99). However, despite the increased bioavailability of BH_4_, which is an important cofactor for endothelial nitric oxide synthase activity in the endothelial production of nitric oxide (99), there was no change in the expression and activation of endothelial nitric oxide synthase during sodium restriction (99). In contrast, in cultured bovine endothelial cells, increased bath sodium concentrations were observed to reduce endothelial nitric oxide synthase activity (100). On the other hand, Omland et al. (101) showed that low sodium intake (10 mmol/24 h for 5 days) was not associated with any significant change in the endothelium-dependent vasodilation to methacholine in healthy individuals. This may be because individuals in this study had a lower sodium intake (10 mmol/24 h) compared to other sodium restriction studies (≥20 mmol/24 h). Conversely, in individuals with or without diabetes, García-Ortiz et al. (102) demonstrated a *J*-shaped relationship between quartiles of sodium intake with arterial stiffness parameters and carotid intima-media thickness, which is a commonly used biomarker for arteriosclerosis and future cardiovascular disease risk (103). The discrepancy in results suggests that more studies are required to investigate the association between dietary sodium intake and vascular endothelial function.

### Effects of Sodium Intake on Immune Function

Sodium intake has been proposed to have effects on both the innate and adaptive immune system (104–109), which may impact on atherosclerosis and cardiovascular morbidity and mortality. Atherosclerosis is a major component in the pathogenesis of cardiovascular disease (90) and consists of chronic low-grade inflammation and atherogenesis (110). Oxidized low density lipoproteins involved in atherogenesis (111) are proposed to be one of the leading antigens involved in mediating T cell infiltration into atherosclerotic plaques (110). This is predominated by CD4^+^ T helper (Th) cells, which predominantly have a Th1 phenotype (110, 112, 113). Th1 cells are pro-inflammatory cells which activate pro-inflammatory macrophages and cytolytic CD8^+^ T cells (110). Moreover, the cytokines produced by Th1 cells, especially IFNγ, are proposed to promote atherogenesis through the activation of macrophages, endothelial cells, and smooth muscle cells (114). Additionally, IFNγ also impairs cholesterol efflux and weakens the fibrous cap to destabilize atherosclerotic plaques (114). On the other hand, the role of Th2 cells in atherosclerosis is less clear (110, 113). Despite the suggestion that Th2 cells are antiatherogenic (110, 112, 114), the IL-4 cytokine produced by these cells has controversial effects (110, 112). Studies have shown that IL-4 could have deleterious or no effect on atherosclerosis (115, 116). Therefore, the role of IL-4 in atherosclerosis needs to be elucidated. Th17 cells have been demonstrated to be present in atherosclerotic plaques and may have a pathogenic role in atherosclerosis because they are considered to be highly pro-inflammatory (110). The IL-17A cytokine produced by Th17 cells exerts its pro-inflammatory effects through the recruitment of pathogenic macrophages to the region of inflammation (117) and is also an important mediator of angiotensin II-induced hypertension (105, 118). Conversely, regulatory T (Treg) cells are proposed to be protective in atherosclerosis (110, 119) because they produce anti-inflammatory cytokines such as IL-10 and TGFβ and suppress immune responses through direct and indirect mechanisms (105, 107, 110).

In addition, the innate immune system has also been proposed to be involved in atherosclerosis. Classical lipopolysaccharide (LPS)-induced M1 macrophages are pro-inflammatory and produce pro-inflammatory cytokines, which promote endothelial dysfunction, destabilization of atherosclerotic plaques in advanced atherosclerosis and thrombus formation in acute coronary syndromes (117), and direct the differentiation and proliferation of Th1 and Th17 cell subpopulations (120). On the other hand, alternatively activated M2 macrophages are non-inflammatory and produce anti-inflammatory cytokines such as IL-10 and TGFβ, which inhibit the recruitment of inflammatory cells and production of pro-inflammatory cytokines (117). However, M2 macrophages may have some pro-atherogenic effects because they can produce IL-4 (117). In addition to the controversy mentioned previously, IL-4 also promotes CD36 expression, which is known to be involved in the uptake of oxidized low density lipoproteins and foam cell formation (114).

High sodium intake might be associated with an imbalance in immune homeostasis, with a predisposition toward a pro-inflammatory state (104–109). During high sodium intake, plasma sodium levels are not increased due to the tight regulation of plasma electrolytes by the kidneys (104). However, sodium can accumulate in the skin and muscles *via* a renal-independent mechanism, resulting in an approximate 40mM (40 mmol/L) increase in interstitial sodium compared to plasma in rodents (104). Given that lymphoid tissues have higher osmolarity, the increase in osmolarity in the interstitial compartments can have effects on immune cells in these regions (104, 109, 121). Increased sodium chloride concentrations *in vitro* (increased by 40 mmol/L) have been demonstrated to promote activation of Th17 cells and M1 (pro-inflammatory) macrophages and blunt activation of M2 (non-inflammatory) macrophages and Treg cells (104, 121). The activation and responses of activated M1 macrophages were increased in the presence of higher sodium chloride concentrations *in vitro* (increase by 40 mmol/L) (104, 121). On the other hand, the activation and ability of M2 macrophages to suppress CD4^+^ and CD8^+^ T cell proliferation were blunted (104). However, this was not associated with increased polarization toward the M1 phenotype in macrophages (104). In addition, a study in 20 healthy non-smoking individuals showed that short-term high sodium intake (sodium intake of 256 mmol/24 h for 7 days) was associated with the induction of pro-inflammatory intermediate (CD14^++^CD16^+^) monocytes and increased production of intracellular reactive oxygen species (122). Moreover, it was also associated with increased monocyte–platelet aggregates (122), which play an important role in thrombotic disorders (123). Conversely, short-term low sodium intake (sodium intake of 85.5 mmol/24 h for 7 days) was associated with a regression of these changes (122). This suggested that high sodium intake may be associated with increased inflammation and thrombosis and low sodium intake may reverse these changes. However, the findings of this study may not be applicable to long-term changes in sodium intake. Yi et al. (106) addressed this by investigating the effects of long-term high and low sodium intakes on immune function in six healthy men. In this study, long-term high dietary sodium intake (203 mmol/24 h for 50 ± 10 days) was associated with increased monocyte count (106). Conversely, long-term lower dietary sodium intake (sodium intake of 153 mmol/24 h for 50 ± 10 days and 102 mmol/24 h for 50 ± 10 days) was associated with reduced production of pro-inflammatory cytokines IL-6 and IL-23 and increased production of anti-inflammatory cytokine IL-10 (106).

Serum-and-glucocorticoid-regulated kinase 1 (SGK1) acts as a mediator of sodium homeostasis by regulating sodium reabsorption through activation of epithelium sodium channels (ENaC) in the kidneys (108). Expression of this enzyme can be induced by exogenous sodium and it is involved in impairing Treg function and enhancing Th17 cell differentiation during increased sodium chloride concentrations *in vitro* and during increased sodium intake *in vivo* (107, 108). Increases in sodium chloride concentrations promoted SGK1 and IL-23 receptor expression and Th17 cell differentiation in a SGK-1-dependent manner *in vitro* (40 mmol/L sodium chloride) and *in vivo* (mouse models) (108). This was supported by another study, which demonstrated that increased sodium chloride concentrations (increased by 40 mmol/L) *in vitro* promoted the stable induction of human and murine Th17 cells through the activation of the p38/MAPK pathway involving nuclear factor of activated T cells 5 (NFAT5) and SGK1 (109). Moreover, Th17 cells induced under increased sodium chloride conditions exhibit a pathogenic phenotype associated with increased expression of pro-inflammatory cytokines including IL-17A (109). In addition, Treg cells are also affected by increases in sodium chloride concentrations. Hernandez et al. (107) demonstrated that increased sodium chloride concentrations *in vitro* (increased by 40 mmol/L in human and murine Treg cells) and *in vivo* (8 and 1% sodium chloride in immune-deficient NOD-scid IL2Rg^null^ mouse models) significantly impaired the suppressive function of Treg cells and promoted a pro-inflammatory Th1-type effector phenotype associated with a SGK1-dependent increase in IFNγ secretion in Treg cells. This illustrated that T cell populations can exhibit plasticity depending on the microenvironment and highlights the importance of environmental influences on T helper cell polarization.

Most of the studies investigated the effect of high sodium intake on immune function (104, 105, 107–109). There is a paucity of data showing the effect of sodium restriction on immune cells. In addition, the studies were mainly performed *in vitro* or in experimental models where genetic modification is possible (104, 107–109). The evidence in human studies is insufficient and circumstantial (110). Moreover, the focus of many studies was on autoimmune diseases (107, 109). Although atherosclerosis involved chronic low-grade inflammation (110), findings from these studies may not be completely translational to cardiovascular disease. Therefore, more studies are required to investigate the effect of sodium restriction on immune cells and its impact on subsequent development of cardiovascular disease in humans.

There is ample evidence demonstrating that the effects of sodium extend beyond blood pressure and contribute to cardiovascular health. As a result, it may not be suitable to derive an association between sodium intake and cardiovascular outcomes based on blood pressure alone. Therefore, studies that explore the association between sodium intake and cardiovascular morbidity and mortality are important.

#### Association between Sodium Intake and Mortality

Despite overwhelming evidence supporting the association between high sodium intake and elevated blood pressure (6), the evidence demonstrating a relationship between dietary sodium intake and cardiovascular outcomes is limited and mostly indirect (124). Some studies proposed that there was no association between dietary sodium intake and cardiovascular morbidity and mortality (125, 126). A study of older adults in the general population showed that sodium intake was not associated with 10-year mortality, incident cardiovascular disease, and incident heart failure (125). In addition, there was no strong evidence that sodium restriction reduced all-cause mortality and cardiovascular disease morbidity in individuals with hypertension or normotension (126).

**Figure 1:**
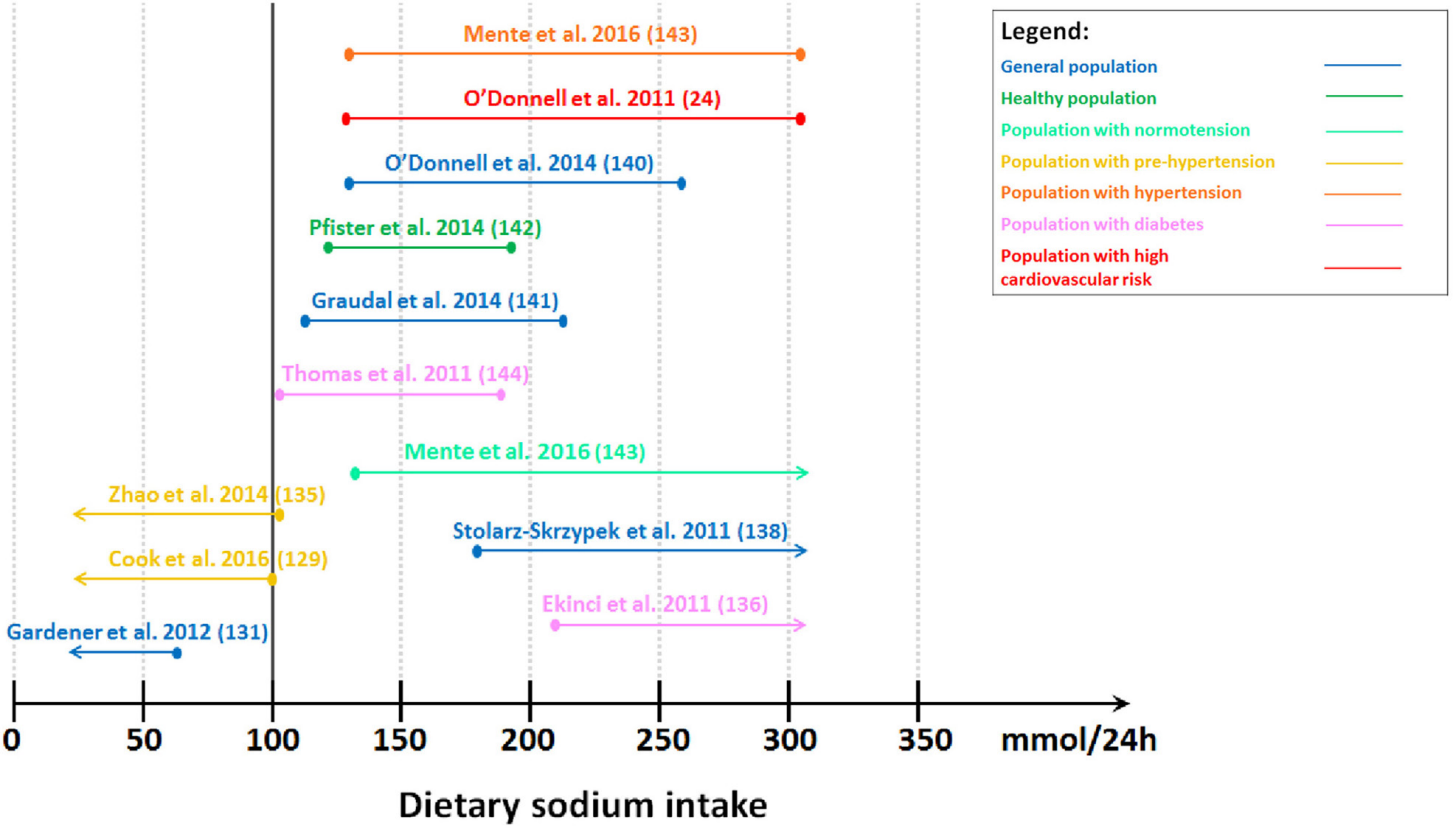
**Level of estimated sodium intake associated with lower cardiovascular morbidity and mortality. Studies showing lower cardiovascular morbidity and mortality with lower sodium intake**. Gardener et al. (131): lowest tertile (≤65 mmol/24 h). Cook et al. (129): lowest quartile (<100 mmol/24 h). Zhao et al. (135): lower median (<102 mmol/24 h). Studies showing ***U***-shaped or ***J***-shaped association. Thomas et al. (144): second tertile (102–187 mmol/24 h). Graudal et al. (141): second tertile (115–215 mmol/24 h). Pfister et al. (142): second–fourth quintile (128–190 mmol/24 h). O’Donnell et al. (140): second–third quintile (130–260 mmol/24 h). O’Donnell et al. (24): third–fifth septile (130–304 mmol/24 h). Mente et al. (143): second–fifth sextile (130–304 mmol/24 h) for individuals with hypertension. Studies showing lower cardiovascular morbidity and mortality with higher sodium intake. Mente et al. (143): second–seventh sextile (≥130 mmol/24 h) for individuals with normotension. Stolarz-Skrzypek et al. (138): highest tertile (≥178 mmol/24 h). Ekinci et al. (136): highest tertile (>208 mmol/24 h).

In contrast, observational studies and meta-analyses suggested that high sodium intake increased adverse cardiovascular outcomes (79, 127–135) (Figure 1). Tuomilehto et al. (134) reported that higher sodium intake independently predicted increased mortality and risk of coronary heart disease in Finnish individuals aged 25–64 years old. It was observed that the association was more prominent in men who had high BMI (≥27 kg/m^2^) (134). This finding was supported by studies conducted in the general population aged 25–74 years old (127) and in individuals with prehypertension aged 45–75 years old (systolic blood pressure of 120–139 mmHg or diastolic blood pressure of 80–89 mmHg) (135). Moreover, higher sodium intake was shown to be associated with increased risk of stroke and stroke mortality in the general population (131, 133). In addition, a meta-analysis of prospective studies in adults also demonstrated an association between higher sodium intake and increased risk of stroke and total cardiovascular disease (132). On the other hand, a study projected substantial benefits of dietary sodium restriction on reducing cardiovascular outcomes based on the effects of sodium restriction on blood pressure reduction (128). Despite the inappropriate assumption of a linear relationship between sodium intake and blood pressure and between blood pressure and cardiovascular events in this study (128), there are other studies supporting the reduction of cardiovascular morbidity and mortality during sodium restriction (128, 130). The observational follow-up study of Trials of Hypertension Prevention (TOHP) phase I and II showed that lower average sodium intake was associated with reduced mortality in individuals with prehypertension aged 30–54 years old, suggesting that sodium restriction may reduce long-term risk of cardiovascular events in these individuals (129, 130). This relationship was observed even in the lowest range of sodium intake (<100 mmol/24 h) (129). Additionally, Aburto et al. (79) also reported reduced risk of stroke and fatal coronary heart disease with lower sodium intake in adults.

In contrast, we demonstrated that low sodium intake was associated with higher cardiovascular morbidity and mortality in individuals with type 2 diabetes (136, 137) (Figure 1). This association was also observed in the general population even after excluding individuals with pre-existing cardiovascular disease (138, 139) (Figure 1). Conversely, other studies which followed suggested a *J*-shaped (24, 140) or *U*-shaped (141–144) relationship (Figure 1). O’Donnell et al. (140) reported a *J*-shaped association between cardiovascular events and sodium intake measured by estimated sodium excretion, with a lower risk of death and cardiovascular events when estimated sodium intake was between 3 and 6 g/day (130–261 mmol/24 h) in the general population aged 35–70 years old in the Prospective Urban Rural Epidemiology (PURE) study. Another study in individuals at high risk of cardiovascular disease supported this by showing that the risk of cardiovascular events was higher with estimated sodium intake less than 3 g/day (130 mmol/24 h) and greater than 7 g/day (304 mmol/24 h) (24). In contrast, Pfister et al. (142) showed a *U*-shaped association between sodium intake measured by 24-h urinary sodium excretion and heart failure in the general population. This *U*-shaped association was also observed between sodium intake and all-cause mortality in individuals with type 1 diabetes without end-stage renal failure (144). A meta-analysis demonstrated that usual sodium intake (115–215 mmol/24 h) had the lowest risk of all-cause mortality and cardiovascular disease events with no difference between the higher and lower end of this “normal” range in adults (141). Mente et al. (143) supported this by demonstrating a *U*-shaped association between sodium intake and cardiovascular events and mortality in the general population. However, this relationship was only observed in individuals with hypertension. In individuals with hypertension, a lower sodium intake of less than 3 g/day (130 mmol/24 h) and higher sodium intake of at least 7 g/day (304 mmol/24 h) were associated with a higher risk of cardiovascular events and death than a sodium intake of 4–5 g/day (174–217 mmol/24 h). Conversely, in normotensive individuals, only lower sodium intake was associated with increased risk of cardiovascular events and death (143). This suggests that the current recommendations to reduce sodium intake may need to be revaluated.

#### Differences in Effects of Dietary Sodium Intake on Health in the Diabetes Population Compared to the General Population

**Figure 2:**
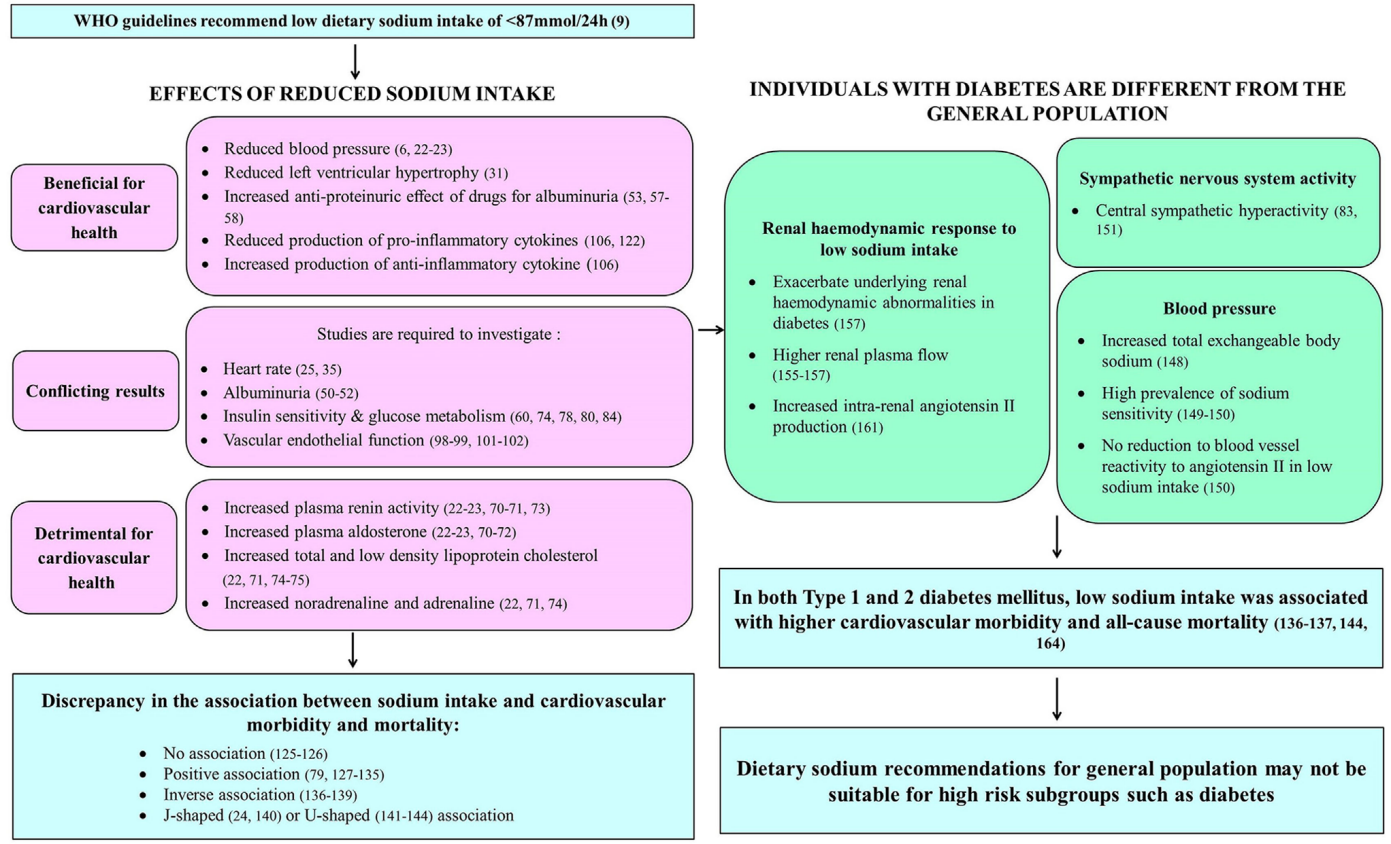
**Summary of effects of dietary sodium intake on systems contributing to cardiovascular health in those with and without diabetes**.

Cardiovascular disease accounts for up to 80% of deaths in individuals with diabetes (145). Since diabetes is associated with multiple cardiovascular disease risk factors, including hypertension, dyslipidemia, microalbuminuria, and left ventricular hypertrophy (145), which in turn are associated with dietary sodium intake, the impact of sodium intake on cardiovascular morbidity and mortality in diabetes may differ from the general population (Figure 2). Hypertension is present in approximately 70% of individuals with type 2 diabetes (146) and this further increases cardiovascular disease risk in these individuals (147). Individuals with diabetes were shown to have significantly increased total exchangeable sodium compared to normal individuals (148). This excess body sodium may play an important pathogenetic role in maintaining diabetes-associated hypertension (148). In addition, a high prevalence of sodium sensitivity has been reported in individuals with type 1 diabetes (149) and in individuals with hypertension and type 2 diabetes (150). However, in contrast to individuals without diabetes, low sodium diets did not reduce the reactivity of blood vessels to angiotensin II, indicating that sodium restriction may be less effective for blood pressure control in individuals with hypertension and type 2 diabetes compared to individuals without diabetes (150).

In addition, central sympathetic hyperactivity was reported in individuals with type 2 diabetes, with the greatest sympathetic hyperactivity seen in those with concurrent essential hypertension (83, 151). Type 2 diabetes is associated with hyperinsulinemia (78), which is secondary to insulin resistance (145). Hyperinsulinemia has been known to increase sympathetic output through the sympathoexcitatory effects of increased insulin (152). Moreover, Huggett et al. (83) have demonstrated an association between increased sympathetic nervous system activity and increased insulin levels. Therefore, the excessive sympathetic nervous system activation observed in type 2 diabetes may be attributed to increased insulin levels (151). This sustained over-activation of the sympathetic nervous system may contribute to the increased cardiovascular morbidity and mortality associated with type 2 diabetes (83). As such, the effects of dietary sodium intake on sympathetic nervous system activity may play a more important role in the cardiovascular health of individuals with diabetes.

Diabetic nephropathy is the leading cause of end-stage renal disease worldwide (153, 154). Despite the lack of understanding of the pathogenesis of diabetic nephropathy, it was observed that the occurrence of glomerular hyperfiltration in early diabetes contributes to it (154, 155). Studies have shown increased glomerular filtration rate and renal plasma flow in individuals with type 1 diabetes and higher effective renal plasma flow in individuals with type 2 diabetes compared to individuals without diabetes (155–157). In addition, the renal hemodynamic response to variations in dietary sodium intake may be different in diabetes compared to the general population (154). This is due to primary tubular hyperresorption in early diabetes and the corresponding normal physiologic action of the tubuloglomerular feedback system (154, 158). Glucose and sodium in the renal proximal tubule lumen are reabsorbed *via* sodium-glucose cotransporters (SGLT) in the luminal side of proximal tubule epithelial cells (159). This is driven by the sodium gradient within these cells, which is generated by the sodium-potassium ATPase pump in the basolateral membrane, thereby facilitating the transport of glucose against a concentration gradient (159). The tubuloglomerular feedback system theory stipulates that increased glucose and sodium reabsorption in the proximal tubules of the kidney during primary tubular hyperresorption in early diabetes leads to reduced sodium concentration at the macula densa, which in turn leads to afferent arteriolar vasodilation to increase glomerular filtration rate, resulting in glomerular hyperfiltration (154). In individuals with type 1 diabetes, sodium restriction was demonstrated to exacerbate the underlying renal hemodynamic abnormalities seen in early disease, including increased glomerular filtration rate and decreased renal vascular resistance (157). In contrast to the increase in effective renal plasma flow associated with higher sodium intake in individuals without diabetes (160), individuals with type 1 diabetes were observed to have significantly higher renal plasma flow during extreme sodium restriction (157). In addition, individuals with type 2 diabetes were reported to have low baseline plasma renin activity (161), which may be associated with an increased risk of developing aldosterone escape (64). They had a heightened renal vasodilator response to angiotensin receptor blockers (irbesartan) despite a limited increase in plasma renin activity during extreme sodium restriction (161). Therefore, it was proposed that plasma renin might not reflect intrarenal renin levels in type 2 diabetes (161). Moreover, the increase in plasma renin activity induced by the angiotensin receptor blocker suggested that there might be increased intrarenal angiotensin II production suppressing plasma renin activity in type 2 diabetes (161).

Given these differences between people with diabetes and the general population (Figure 2), cardiovascular outcomes associated with variations in dietary sodium intake in diabetes may not be the same as that expected of the general population. Therefore, the paradoxical relationship between low sodium intake and higher cardiovascular morbidity and mortality should not be disregarded, especially in patient populations with specific clinical conditions (162).

In individuals with type 1 diabetes, both high and low dietary sodium intakes were shown to be associated with increased all-cause mortality (144). Moreover, in these individuals, there was also an inverse association between sodium intake and the development of end-stage renal disease (144), which is associated with a significantly increased mortality risk (163). As a result, this could contribute to increased mortality risk during low sodium intake in individuals with type 1 diabetes. Lower dietary sodium intake was observed to be associated with higher all-cause and cardiovascular mortality in individuals with type 2 diabetes (136, 164). This was supported by the finding that lower 24-h urinary sodium excretion over time was also associated with increased all-cause mortality in these individuals (137). Therefore, this suggests that the low dietary sodium intake recommendations for the general population may not be suitable for high-risk subgroups, such as individuals with type 1 diabetes or type 2 diabetes.

## Conclusion

Dietary sodium intake recommendations support sodium restriction based on previous evidence suggesting a reduction in blood pressure. It was proposed that this would be associated with a subsequent reduction in cardiovascular morbidity and mortality. However, increasingly, it is now being understood that sodium intake has other pleiotropic effects that affect cardiovascular health, highlighting that the association between sodium intake and cardiovascular outcomes cannot be based on blood pressure alone. Therefore, current dietary sodium intake guidelines have been revised since the IOM reported that there was no clear benefit or harm of sodium restriction to less than 100 mmol/24 h in 2013. However, recently, the current dietary sodium guidelines have been challenged because there is emerging evidence to suggest an associated increase in morbidity and mortality with lower dietary sodium intake in high-risk groups, including those with diabetes. These studies suggest that the current dietary guidelines may be too strict; therefore, they may not be suitable and may need further revision. However, there is a lack of data from randomized controlled trials to determine the optimal level of dietary sodium intake for specific populations. In addition, there is a paucity of data with a lack of randomized controlled trial data in humans to explain the possible mechanisms contributing to the adverse outcomes associated with lower dietary sodium intake in high-risk populations.

## Author Contributions

YK undertook the literature review and wrote the first draft of the manuscript. EE, SB, and GJ planned the study, reviewed, and edited the manuscript.

## Conflict of Interest Statement

The authors declare that the research was conducted in the absence of any commercial or financial relationships that could be construed as a potential conflict of interest.
